# Preparation of Laminated Titanium Matrix Composites with High Strength and Plasticity via Regulating Heat Treatment Processes

**DOI:** 10.3390/ma18071429

**Published:** 2025-03-24

**Authors:** Xiong Zou, Yu Yang, Junliang Liu, Tingting Sun, Fuqin Zhang

**Affiliations:** Powder Metallurgy Research Institute, Central South University, Changsha 410083, China; zouxiong856@163.com (X.Z.); liujunliang2244@163.com (J.L.); sttwly@163.com (T.S.)

**Keywords:** laminated structure, titanium matrix composites, heat treatment, mechanical properties

## Abstract

In order to achieve a balance between the strength and ductility of titanium matrix composites (TMCs), a spray deposition method was employed to deposit carbon nanotubes (CNTs) onto the surface of Ti foil. Subsequently, spark plasma sintering (SPS) at 850 °C and an additional 1 h heat treatment at 880 °C were utilized to fabricate two laminated composites of different composition, namely, CNTs/Ti (SPS) and in situ TiC/Ti (SPS+HT). The microstructure evolution, mechanical properties, and strengthening and fracture mechanisms of laminated composites were systematically studied. The results revealed that after sintering at 850 °C, the reaction between CNTs and the titanium matrix was limited. However, after a 1 h heat treatment at 880 °C, CNTs were completely transformed into TiC, while the titanium matrix remained α phase without undergoing phase transformation. Through rolling and annealing, TiC particles were refined to 500 nm and exhibited a flattened shape. The in situ TiC/Ti layered composite material exhibited a tensile strength (UTS) of 491.51 MPa, which was a 29.63% improvement compared to pure titanium (379.16 MPa), and significantly higher than the UTS of CNTs/Ti samples (419.65 MPa). The primary strengthening mechanism was load transfer strengthening. The elongation (EL) remained at 26.59%, slightly lower than pure titanium (29.15%) and CNTs/Ti samples (27.51%). This can be attributed to the increased connectivity of the matrix achieved through rolling, which enhanced the ability to passivate cracks and prolonged the crack propagation path. This study presents a method for preparing laminated titanium matrix composites with both strength and ductility by controlling the heat treatment process.

## 1. Introduction

Titanium matrix composites (TMCs) have gained increasing attention in the aerospace, automotive, and military industries due to their exceptional specific strength, specific stiffness, outstanding creep resistance, and corrosion resistance [1,2,3]. Commonly used reinforcements in TMCs include TiC [4], SiC [5], TiB [6,7], and Al_2_O_3_ [8]. Previous studies [9,10] have employed casting or powder metallurgy techniques to produce composites with uniformly distributed particle reinforcements. While these composites exhibit significantly improved strength, their toughness and ductility inevitably decrease [11].

Inspired by the structure of natural shells [12], researchers have endeavoured to construct layered composites to address this issue [13,14]. Compared with composite materials reinforced with uniformly distributed particles, the ability of the metal matrix in layered composite to undergo plastic deformation helps to eliminate concentrated high stress and improve ductility [15]. Duan [16] fabricated Ti-(TiBw + La_2_O_3_)/Ti layered composites through powder metallurgy and hot rolling, resulting in a significant increase in elongation. Hao [17] successfully produced CNTs/Ti laminated composites by means electrophoretic deposition, followed by spark plasma sintering at 550 °C, achieving a simultaneous enhancement in strength and plasticity. Tan [18] successfully prepared in situ TiC/Ti layered composites by means of hot-pressing sintering using graphite paper and Ti foil, exhibiting a fracture toughness of 24.78 ± 0.71 MPa·m^1/2^.

CNTs have a range of excellent properties [19,20,21], but research on their use as reinforcements in titanium matrix composites is relatively limited. Therefore, studying CNTs-reinforced laminated titanium matrix composites is highly meaningful. However, when CNTs are used as reinforcements in titanium matrix composites, a challenge arises: the sintering temperature cannot be too high, as CNTs will undergo significant or complete transformation into TiC [18]. Conversely, at lower sintering temperatures, although the reaction between CNTs and the matrix is suppressed, the interface bonding between CNTs and the titanium matrix is weak, resulting in numerous defects such as porosity within the material. Thus, it becomes difficult to fully utilize the reinforcing effect of CNTs. Considering the high strength and modulus, similar thermal expansion coefficient, and good interface bonding with the titanium matrix of TiC [22,23], this study takes a different approach by promoting the reaction between CNTs and the titanium matrix to prepare in situ TiC/Ti layered composites. This paper systematically investigates the microstructure, mechanical properties, and strengthening and toughening mechanisms of pure titanium, CNTs/Ti, and CNTs/Ti+HT specimens. The goal is to adjust the heat treatment process to prepare laminated titanium matrix composites with both strength and plasticity.

## 2. Experimental Procedures

### 2.1. Preparation of Laminated Composites

**Figure 1:**
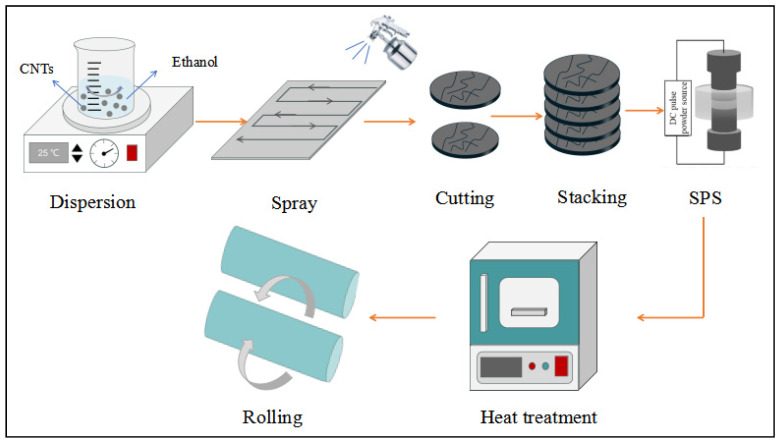
The schematic diagram of the fabrication of laminated composites.

Ti foils, with a thickness of 50 µm, were supplied by Beijing Qianshuo Nonferrous Metal Co., Ltd., Beijing, China and acid-washed before use with a ratio of V(HF):V(HNO_3_) = 1:3. Multi-walled carbon nanotubes (hereinafter referred to as CNTs, 95% purity, 8–15 nm in diameter, and 5 µm in length) were provided by Chengdu Organic Chemistry Co. Ltd., Chengdu, China. The experimental process is shown in Figure 1. Firstly, CNTs were added into the alcohol solution, and ultrasonic dispersion and magnetic stirring were carried out for 2 h. Then, the dispersed solution was added into the spray bottle and uniformly sprayed on the preheated Ti foils, and then the Ti foils were cut and stacked to make specimens containing CNTs with a mass fraction of 0.15%. Subsequently, SPS at 850 °C, 40 MPa, and 10 min was performed. A set of CNTs/Ti composites after SPS was heated in a vacuum at 880 °C for 1 h and then cold-rolled at 60% deformation (40% deformation in the first pass and 10% in each subsequent pass) and held at 600 °C for 0.5 h. As a comparison, the same process was applied to pure Ti foils without added CNTs. Finally, after rolling and annealing, each specimen was subjected to tensile testing.

### 2.2. Characterization

The microstructure of the specimens was observed using scanning electron microscopy (SEM, JEOL JSM-7500F, Tokyo, Japan). The elemental contents and distributions were evaluated by means of energy dispersive spectroscopy (EDS, Oxford X-Max 50, Oxford, UK). X-ray diffraction (XRD, Rigaku SmartLab SE, Tokyo, Japan), instrumented by Cu-Kα radiation in a 2θ range of 30–90 with a scan speed of 1.5°/min, was used for the phase identification. The texture and grain size of the specimens was measured using electron backscattered diffraction (EBSD) employed in the SEM (Zeiss Gemini 500, Oberkochen, Germany). Focused ion beam (FIB, Tescan AMBER GMH, Brno, Czech), which is suitable for the treatment of TiC/Ti composite without structural damages, was used to prepare samples for the characterization of the TiC/Ti interface of the composites. The high-resolution transmission electron microscopy (HRTEM) images and selected area electron diffraction (SAED) patterns were obtained by a transmission electron microscope (TEM, Thermo FEI Talos F200X, Waltham, MA, USA).The tensile strength of the specimens was measured using an universal material testing machine (Instron3369, Canton, MA, USA) with a tensile speed of 1 mm/min. The parallel length of the tensile samples was 15 mm, the width was 6 mm, and the thickness was about 2.5 mm. The tensile test was repeated three times for each case. 

## 3. Results and Discussion

### 3.1. Microstructure

**Figure 2:**
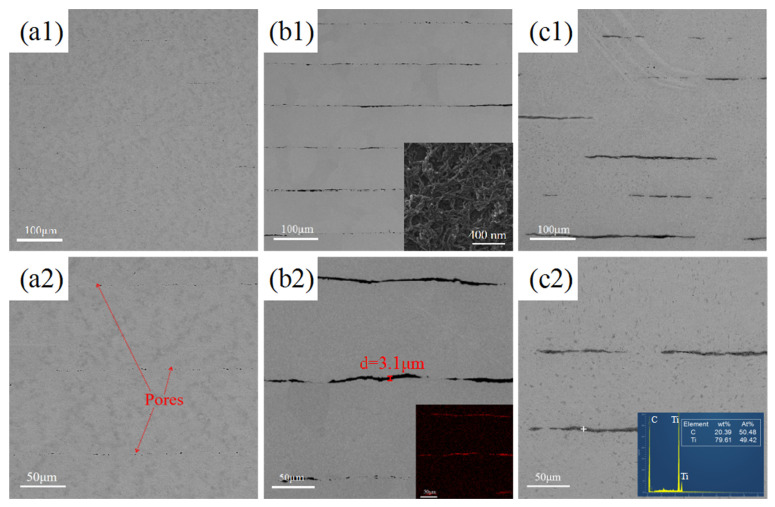
SEM images and corresponding energy spectrum. (**a1**,**a2**) Ti; (**b1**,**b2**) CNTs/Ti; (**c1**,**c2**) CNTs/Ti+HT.

Figure 2 shows SEM images of different specimens after SPS. As shown in Figure 2(a1,a2), the interfaces of the layers of pure titanium specimens are fused together after sintering, but a small amount of porosity exists between the layers due to the interfaces not being completely fused at lower sintering temperatures, which can be improved by the subsequent rolling treatment. The boundary between the Ti layers in the CNTs/Ti specimen can be clearly observed through Figure 2(b1,b2). The additional image in Figure 2(b1) shows the deposition morphology of CNTs on the titanium foil. From this additional image, it can be seen that CNTs are evenly distributed on the surface of the titanium foil, with less aggregation. According to Figure 2(b2), the average thickness of CNTs is 3.1 μm, and the black material distributed between the layers was determined to be elemental C by EDS, which is unreacted CNTs. At the interface, due to the presence of CNTs preventing the fusion of the titanium substrate between the layers, the layers were independent of each other and had very poor connectivity, and at the same time, due to the uneven thickness of the sprayed CNTs, the interface became uneven. As shown in Figure 2(c1,c2), there is a grey lamellar material at the interface of CNTs/Ti+HT specimen, in which there are some tiny holes. The EDS at this point can identify the gray lamellar material is TiC. Mu [24] investigated the interaction of Ti with graphene nanosheets and elucidated in detail the growth process of in situ TiC, which is divided into three stages: initial formation, rapid densification, and complete densification of the TiC layer. The in situ TiC generated from CNTs in this study follows a similar growth mechanism: TiC cores grow parallel to the CNTs/Ti interface until a continuous TiC layer is formed; When the outer TiC layer reaches a dense state, carbon atoms in CNTs pass through the TiC layer to reach the Ti matrix and undergo in situ reactions, causing TiC to continue growing until all CNTs are transformed into in situ TiC; The pores in TiC lamellae are caused by the Kirkendall effect.

**Figure 3:**
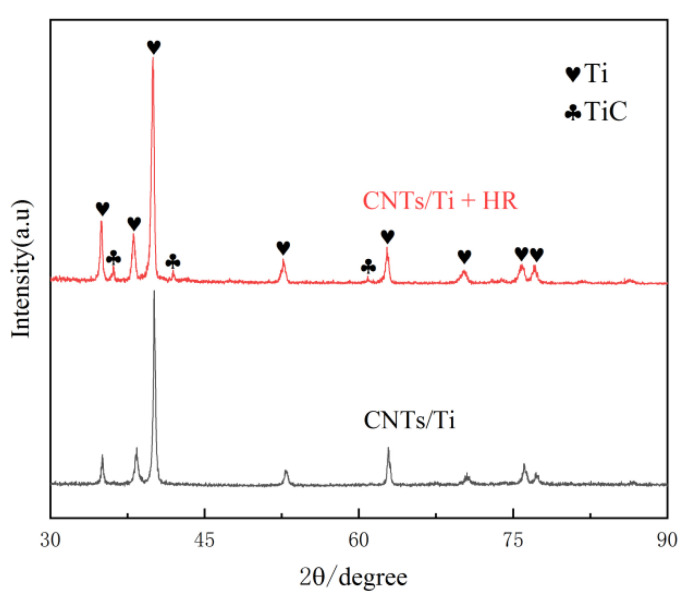
XRD patterns of CNTs/Ti and CNTs/Ti+HT.

Figure 3 shows the XRD patterns of CNTs/Ti and CNTs/Ti+HT samples. It can be seen that no diffraction peaks of TiC appeared in the CNTs/Ti specimens after SPS, indicating the low content of TiC generated during the sintering process, whereas diffraction peaks of TiC appeared in the CNTs/Ti+HT specimens, indicating the massive transformation of CNTs into TiC after heat treatment. The results of the XRD analysis are in full accordance with the SEM conclusions in Figure 2. Choi [25] studied the reaction between Ti and C, and he found that the TiC phase can form at temperatures as low as 685 °C. Dong [26] investigated the reaction temperature between CNTs and Ti, demonstrating that Ti and CNTs have completely reacted at 900 °C to form TiC.

**Figure 4:**
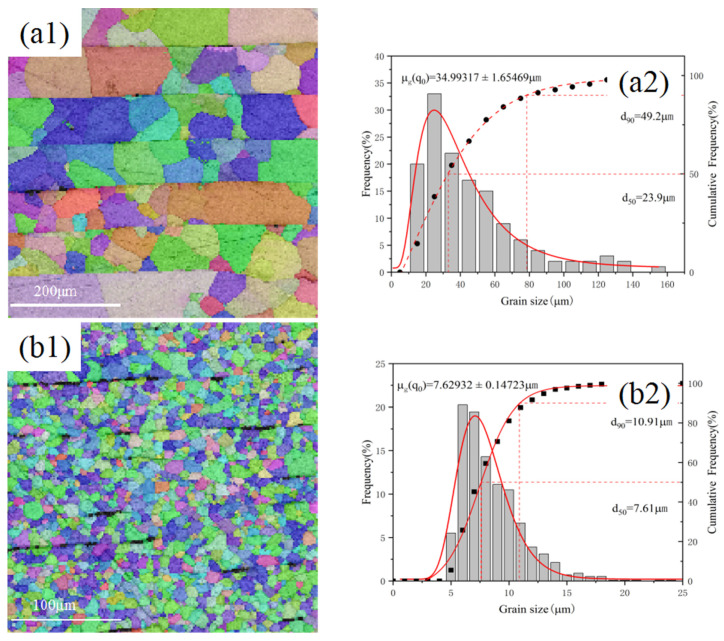
EBSD analysis and corresponding grain size distributions. (**a1**,**a2**) CNTs/Ti+HT; (**b1**,**b2**) CNTs/Ti+HT after rolling and annealing.

To further investigate the microstructural changes in the specimens, EBSD analysis was carried out on the heat-treated and cold-rolled–annealed specimens, respectively, and the grain size distribution was calculated accordingly. As shown in Figure 4(a1), the titanium matrix after heat treatment exhibits large grains, where the thickness of a single grain is often comparable to the layer thickness. Due to the presence of the TiC layer, no grain growth was observed across the interfacial layer. Figure 4(a2) shows that the average grain size of the composite material is 35.0 μm, and the grain size corresponding to a cumulative frequency of 50% is 23.9 μm, indicating a significant variation in grain size within the matrix. As shown in Figure 4(b1), during repeated rolling, a large number of grains are deformed and broken, and defects such as pores and microcracks in the matrix and at the interface are bridged. Due to the large amount of strain energy accumulated in the matrix, recrystallisation occurs and a large number of fine equiaxial grain are generated during the annealing process. As rolling improves the distribution of the TiC layers, the TiC layers become discontinuous and the interlayer interface of the titanium matrix gradually fuses. Notably, the grain size in the fusion zone is consistent with that in the interface region. Figure 4(b2) shows that the average grain size of the matrix is 7.62 μm, and the grain size corresponding to a cumulative frequency of 50% is 7.61 μm, which is close to the average grain size, suggesting a relatively concentrated distribution of grain sizes.

**Figure 5:**
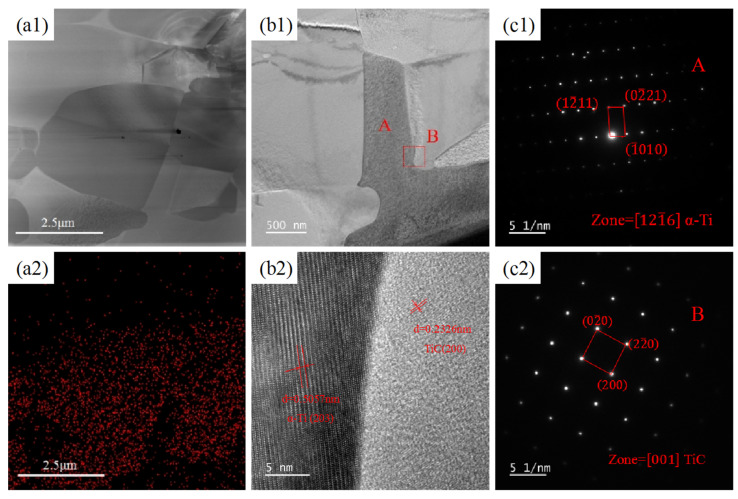
TEM images of CNTs/Ti+HR: (**a1**,**a2**) dark field image and associated EDS maps for C; (**b1**,**b2**) bright field image and corresponding HRTEM in the red box area; (**c1**,**c2**) SAED pattern of region A and B in (**b1**).

Figure 5 presents the TEM images of the CNTs/Ti layered composites after being held at 880 °C for 1 h. According to the distribution of carbon in Figure 5(a2), it can be initially judged that the grey particles enriched with carbon in Figure 5(a1) are TiC, with particle sizes ranging from 1 to 3 μm, and the distribution is relatively concentrated to form an irregular lamellar layer. To further determine the composition of these particles and whether any CNTs remain at the interface, SAED and HRTEM were performed on the box-marked area in Figure 5(b1). According to the results shown in Figure 5(b2,c1,c2), CNTs seem to have completely reacted, and no residual CNTs are observed. The regions A and B shown in Figure 5(b1) are α-Ti and TiC, respectively, verifying the earlier judgement that the carbon-rich particles are TiC. The phase transition point of pure titanium is 882.5 °C [27], and the reason for choosing 880 °C for heat treatment in this study is double. Firstly, CNTs can be completely converted into TiC, and secondly, the phase transition of titanium substrate during the heating process can be avoided. If the temperature exceeds the phase transition point, the β phase will be generated, and during cooling, the newly formed α phase will grow into Widmanstätten structures along β grain boundaries [28], reducing the plasticity of the material and making it unfavourable for subsequent cold rolling processes. 

**Figure 6:**
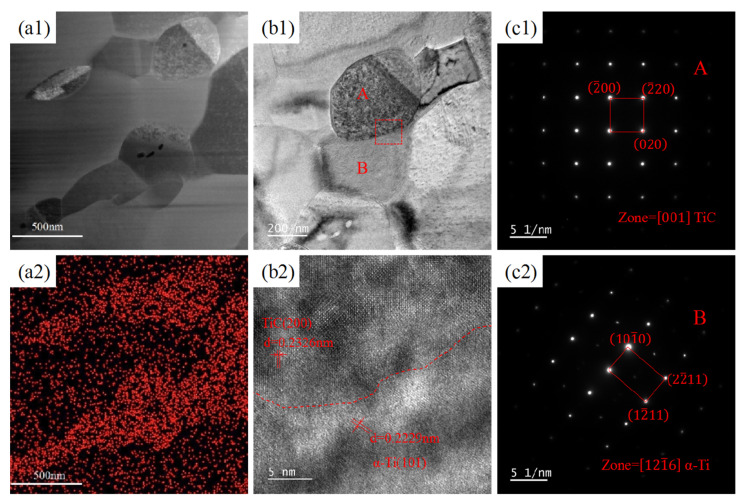
TEM images of CNTs/Ti+HR after rolling and annealing: (**a1**,**a2**) dark field image and associated EDS maps for C; (**b1**,**b2**) bright field image and corresponding HRTEM in the red box area; (**c1**,**c2**) SAED pattern of region A and B in (**b1**).

Figure 6 shows the TEM images of the CNTs/Ti+HR specimen after cold rolling and annealing. It can be seen that the sample is still composed of TiC and α-Ti, in which the TiC particles are flat and connected to each other, with an average size of about 500 nm. During the rolling process, the TiC particles are stretched and deformed along the rolling direction. Larger TiC particles are hard and brittle, easily breaking during the rolling process. After annealing, the broken TiC particles undergo recrystallisation and regenerate into a series of fine flake particles, and ultimately, the rolled TiC particles are significantly finer and arranged in a certain orientation.

### 3.2. Mechanical Properties

**Figure 7:**
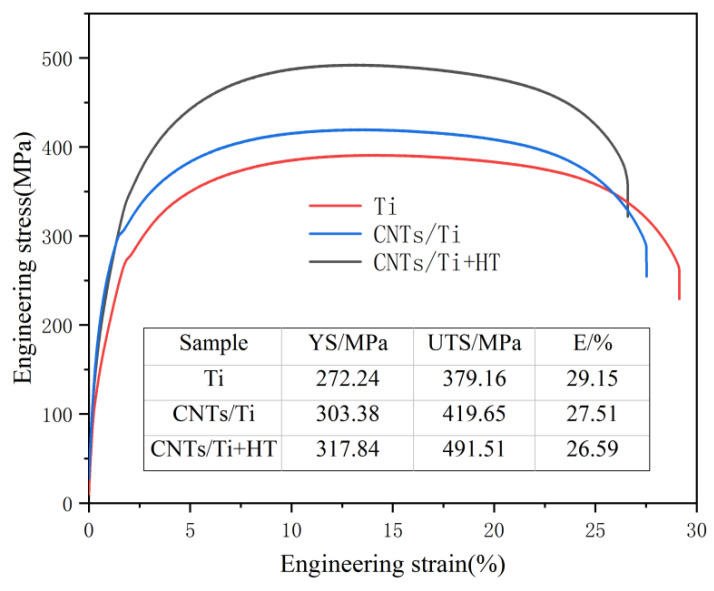
Engineering tensile stress–strain curves of different samples.

Figure 7 shows the tensile engineering stress–strain curve of prepared samples after rolling and tempering at room temperature. The yield strength (YS), tensile strength (UTS), and elongation (EL) of pure titanium are 272.24 MPa, 379.16 MPa, and 29.15%, respectively. The characteristics of high elongation and low strength are difficult to meet the needs of use. For the sample of CNTs/Ti, the yield strength, tensile strength, and elongation are 303.38 MPa, 419.65 MPa, and 27.51%, respectively. The section elongation decreases by 1.64%, but the yield strength and tensile strength increases by 11.4% and 10.67%, indicating that the added CNTs plays a certain role in improving the strength of the material. For the CNTs/Ti+HT sample, the yield strength, tensile strength, and elongation are 317.84 MPa, 491.51 MPa, and 26.59%, respectively. Compared with the pure titanium sample, the yield strength and tensile strength are significantly increased by 16.7% and 29.63%, respectively, while the elongation is only reduced by 2.56%.The performance of CNTs/Ti+HT samples is significantly better than that of CNTs/Ti, achieving a good match between strength and plasticity.

### 3.3. Fracture Morphology

**Figure 8:**
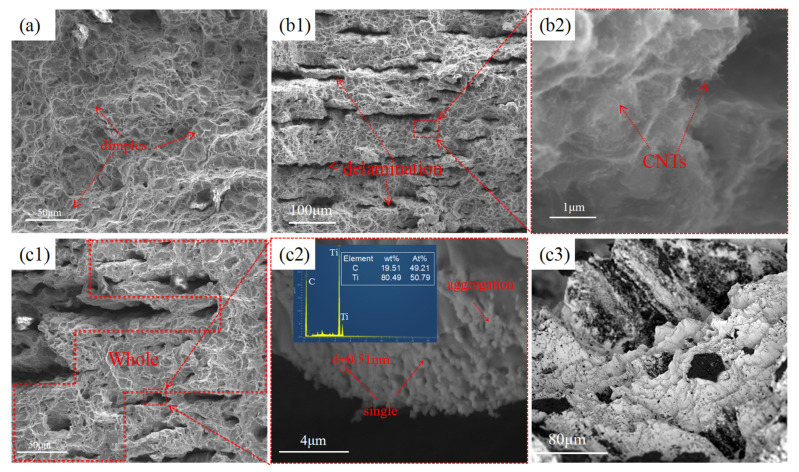
SEM images of fracture morphology of different samples. (**a**) Ti; (**b1**,**b2**) CNTs/Ti; (**c1**–**c3**) CNTs/Ti+HT.

The fracture morphology of the materials after tensile testing is shown in Figure 8. In Figure 8a, the fracture morphology of the pure titanium sample exhibits numerous dimples, indicating a ductile fracture mode. The interfaces within the titanium matrix are well fused, with no observed delamination or cracking. The fracture morphology of the CNTs/Ti sample exhibits distinct interfacial delamination (Figure 8(b1)), and the corresponding enlarged image (Figure 8(b2)) directly shows the existence of CNTs. The CNTs at the interface prevent the fusion of the matrix and reduce overall connectivity. When the cracks extend to the interface containing CNTs, due to insufficient reaction between CNTs and the titanium matrix, strong interfacial bonding is not formed, leading to easy cracking and delamination at the interface. Additionally, the agglomeration of CNTs causes stress concentration during tensile loading, making it easy for cracks to initiate near the CNTs, thereby reducing the material’s plasticity. Figure 8c depicts the fracture surface morphology of the CNTs/Ti+HT sample. Figure 8(c1) shows that delamination also occurs at the interface, but it is significantly reduced compared to the untreated CNTs/Ti specimen, with an improved connectivity of the matrix. Some tiny particles with a diameter of about 0.52 μm can be observed in Figure 8(c2). Combined with the corresponding EDS point scan analysis and TEM results, these particles can be identified as TiC. Unlike the coarse TiC particles, the fine TiC particles have a better coordinated deformation ability with the titanium matrix during tensile loading, which reduces the tendency of cracking at the interface. Moreover, once a crack forms at the interface, the Ti matrix can blunt the crack by creating a plastic zone [29,30]. Figure 8(c3) is an overhead view of the fracture surface. The sawtooth pattern indicates that the main crack has undergone large-scale deflection between layers. Due to the presence of discontinuous TiC layers at the interface, the crack chooses to bypass the TiC layer and continue to extend through the connected titanium substrate, increasing the path of crack propagation. This consumes a significant amount of energy, thus enhancing the material’s toughness. In summary, the reason why the in situ TiC/Ti laminated composite only slightly decreases in ductility while significantly improving its strength compared to the CNTs/Ti laminated composite is the increase in matrix connectivity. This enhanced connectivity can blunt cracks and increase the path of crack propagation.

In this paper, the strength of the laminated composites is higher than that of the pure titanium sample. The main strengthening mechanisms are solution strengthening, fine crystal strengthening, and stress–strain strengthening.

Solid solution strengthening: The solution limit of carbon (C) in α-Ti was 0.05 wt% [31], and the total mass fraction of carbon nanotubes (CNTs) in this paper was 0.15%. However, all of them were distributed at the interface, so the content of carbon at the interface was much higher than 0.15%. According to relevant reports in the literature, when the content of carbon exceeds the solubility limit, the carbon element will precipitate as titanium carbide (TiC). The increase in strength for each 0.01 wt% increase in carbon content can be numerically defined as 7 MPa [32]. Therefore, in this paper, the strengthening of CNTs/Ti and TiC/Ti laminated composites due to solid solution was basically the same, about 35 MPa [33].

Fine grain strengthening: Theoretically, when there is a large number of reinforcement phases in the matrix, the reinforcement phase prevents the fusion and growth of the grains in the titanium matrix, thus refining the grains. However, according to the previous electron backscatter diffraction (EBSD) results, the grain size difference between the interface region and the fusion region is not large, so the contribution of fine grain strengthening to the strength improvement of the material is minimal.

Load transfer strengthening: Strong interface bonding promotes stress transfer from the matrix to the reinforcement phase. For composite materials, load transfer strengthening is the main strengthening mechanism [1]. The added yield strength (Δσ*_LT_*) of composite materials can be evaluated by the shear-lag model and estimated as follows [34,35]:

$$∆\sigma_{LT}=\sigma_{m}\left[\frac{V_{r}\left(S_{eff}+4\right)}{4}+\left(1-V_{r}\right)\right]$$

where *σ_m_* is the 0.2% YS of the Ti matrix, *V_r_* and *S_eff_* are the volume fraction and the effective aspect ratio of the reinforcements, respectively. In this way, the strength of CNTs/Ti composites is higher than that of TiC/Ti composites. However, the experimental results show the opposite, and the difference is obvious, which may be due to the following reasons. (1) The agglomeration of carbon nanotubes resulted in a decrease in the effective binding of CNTs at the interface with the titanium matrix, and a decrease in the number of CNTs that really played the role of load transfer. (2) In in situ TiC/Ti composites, CNTs are all converted to TiC, which increases the contact area between the reinforcement phase and the matrix. Meanwhile, after rolling, TiC particles are not only small but also flat, and their length–diameter ratio increases, thus making in situ TiC/Ti composites have the highest strength.

## 4. Conclusions

The fabrication of CNTs/Ti laminated composites was successfully achieved through spark plasma sintering (SPS). Building upon this, in situ TiC/Ti laminated composites were successfully obtained by means of heating at 880 °C for 1 h. The key findings of this study can be summarized as follows:

1. The chosen heat treatment process proved to be suitable. Heating the material at 880 °C for 1 h effectively prevented phase transformation in the titanium matrix, facilitating the subsequent cold rolling process and enabling the complete conversion of CNTs into TiC as the reinforcing phase. The in situ formed TiC particles exhibited a relatively fine size.

2. Rolling and annealing treatments led to the refinement of the grain size in both the titanium matrix and TiC particles. The grain size of the titanium matrix was reduced from 35 μm to 7.61 μm, while the TiC particle size was refined from 1 to 3 μm to approximately 500 nm. Furthermore, rolling resulted in the flattening of the TiC particles, increasing their aspect ratio and enhancing the load transfer strengthening capability.

3. The tensile strength of the in Situ TiC/Ti laminated composite material reached 491.51 MPa, which was 29.63% higher than that of the pure titanium sample and much higher than that of the CNTs/Ti sample (419.65 MPa). The strengthening mechanism was mainly load transfer strengthening, and the elongation of the two laminated composite materials was not significantly different. This substantial difference highlights the superior performance of the in situ TiC/Ti laminated composites. The in situ TiC/Ti layered composites demonstrated comparable elongation to the CNTs/Ti layered composites. However, there is a huge difference in strength between the two composites. The tensile strength of the two composite materials increased by 29.63% and 10.67% compared to pure titanium samples, respectively. Therefore, the in situ TiC/Ti layered composites have better comprehensive mechanical properties.

## Data Availability

The data presented in this study are available upon request from the corresponding author.
